# Laparoscopic Median Arcuate Ligament Release During Gastric Tube Reconstruction in Subtotal Esophagectomy for a Patient With Esophageal Cancer Complicated by Median Arcuate Ligament Syndrome: A Case Report

**DOI:** 10.7759/cureus.65158

**Published:** 2024-07-22

**Authors:** Jin Yamakado, Ryosuke Shibata, Masato Watanabe

**Affiliations:** 1 Surgery, Fukuoka University Chikushi Hospital, Fukuoka, JPN

**Keywords:** laparoscopic median arcuate ligament release, gastric tube reconstruction, median arcuate ligament syndrome, submucosal invasion, esophageal cancer

## Abstract

A 74-year-old man was diagnosed with squamous cell carcinoma of the lower thoracic esophagus following an upper gastrointestinal endoscopy during a health check-up, which revealed a type 0-IIc tumor. Biopsy confirmed squamous cell carcinoma, with suspicion of submucosal invasion. The patient was referred to our department. Contrast-enhanced computed tomography of the chest and abdomen showed no apparent lymph node or distant metastasis. Severe stenosis at the origin of the celiac artery, likely due to the median arcuate ligament, was observed. No abdominal symptoms were noted at rest or after meals, leading to the diagnosis of thoracic esophageal cancer with asymptomatic median arcuate ligament syndrome. Subsequently, laparoscopic median arcuate ligament release was performed during gastric tube reconstruction in subtotal esophagectomy.

## Introduction

Celiac artery (CA) compression syndrome (CACS) is a condition caused by extrinsic compression at the root of the celiac artery (CA), resulting in stenosis. This condition was first suggested by anatomist Lipshutz [1] in 1917 and first reported clinically by Harjola [2] in 1963. CACS caused by the median arcuate ligament (MAL) is termed MAL syndrome (MALS), which manifests with various symptoms due to diminished blood flow in the regions perfused by the CA. The standard curative treatment for resectable esophageal cancer includes esophagectomy, reconstruction, and perioperative therapy. Despite significant advances in surgical techniques and perioperative management over the past few decades, the attendant complication rates (60%) and perioperative mortality (5%) remain high. Anastomotic leakage, a severe postoperative complication, occurs in 10-21.2% of patients, attributable to impaired tissue perfusion in the proximal part of the gastric tube. Lainas et al. reported that CA stenosis due to MALS, in addition to atherosclerotic CA stenosis, is associated with anastomotic leakage and gastric tube necrosis following Ivor-Lewis esophagectomy [3]. In the reconstruction of a subtotal esophagectomy, a gastric tube is created by dissecting part of the normal stomach and its nutrient vessels and anastomosing it with the cervical esophagus. Compression of the CA by MAL leads to decreased blood flow to the right gastroepiploic artery, the only nutrient vessel in the gastric tube. As a result, it is suggested that the anastomosis between the gastrointestinal tract and the cervical esophagus may induce suture failure due to tissue anastomosis. In the present study, we performed a subtotal esophagectomy for a patient with thoracic esophageal cancer with MALS, and laparoscopic MAL resection was used for the reconstruction of the gastric tube. We report a case in which we succeeded in securing blood flow to the gastrointestinal tract by increasing blood flow to the right gastroepiploic artery via the CA.

## Case presentation

The patient was a 74-year-old man who presented with a chief complaint of abnormality detected during a health check-up at another center. The current medical history included a type 0-IIc tumor in the lower thoracic esophagus detected on upper gastrointestinal endoscopy during a health examination, which was diagnosed as squamous cell carcinoma by biopsy. He was referred to our department upon suspicion of submucosal invasion. The past medical history included hyperuricemia, appendectomy, left inguinal hernia repair (open method), and ascending colon polyp removal (endoscopic submucosal dissection). Physical examination on admission revealed the following: height of 170 cm, weight of 70 kg, and a flat and soft abdomen without tenderness or postprandial symptoms. Post-surgical scars from appendectomy and inguinal hernia repair were observed.

Blood test results are shown in Table 1.

**Table 1:** Blood test results.

Checklist	Numerical value	Unit	Normal level
White blood cells	7200	/μL	3300-8600
Red blood cells	493×104	/μL	Male 435-555×104, Female 386-492×104
Hemoglobin	16.3	g/dL	Male 13.7-16.8, Female 11.6-14.8
Hematocrit	48.7	%	Male 40.7-50.1, Female 35.1-44.4
Platelet	21.8×104	/μL	15.8-34.8×104
Prothrombin time - international normalized ratio	1		0.85-1.15
Prothrombin time	11.3	s	10-13
Activated partial prothrombin time	31.1	s	24-39
Total protein	8.1	g/dL	6.6-8.1
Albumin	4.5	g/dL	4.1-5.1
Total bilirubin	1.4	mg/dL	0.4-1.5
Aspartate transaminase	20	U/L	7-38
Alanine transaminase	17	U/L	4-44
Blood urea nitrogen	16	mg/dL	8-20
Creatinine	1.29	mg/dL	Male 0.65-1.07, Female 0.46-0.79
Estimated glomerular filtration rate	42.7	ml/min/1.73 m^2^	>60
Na	141	mmol/L	137-147
K	4.3	mmol/L	3.5-5.0
Cl	105	mmol/L	98-108
C-reactive protein	0.03	mg/dL	<0.14
Carcinoembryonic antigen	1.6	ng/mL	1.0-5.0
Squamous cell carcinoma antigen	3.5	ng/mL	<1.5

**Figure 1:**
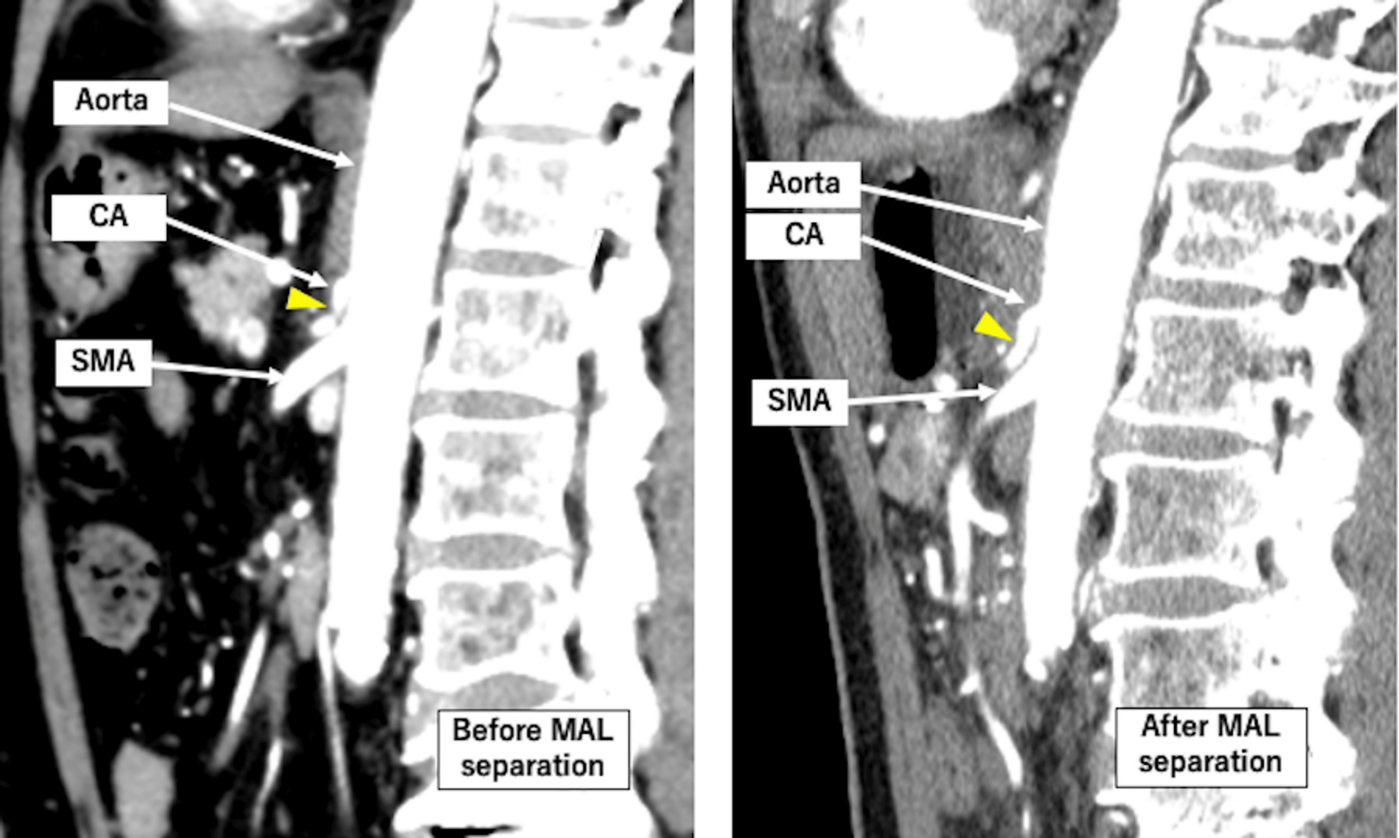
Contrast-enhanced CT of the thorax and abdomen. Sagittal section: (▲) CT images before and after MAL resection are shown. No calcification is present in the CA. (MAL: median arcuate ligament, CA: celiac artery, SMA: superior mesenteric artery)

**Figure 2:**
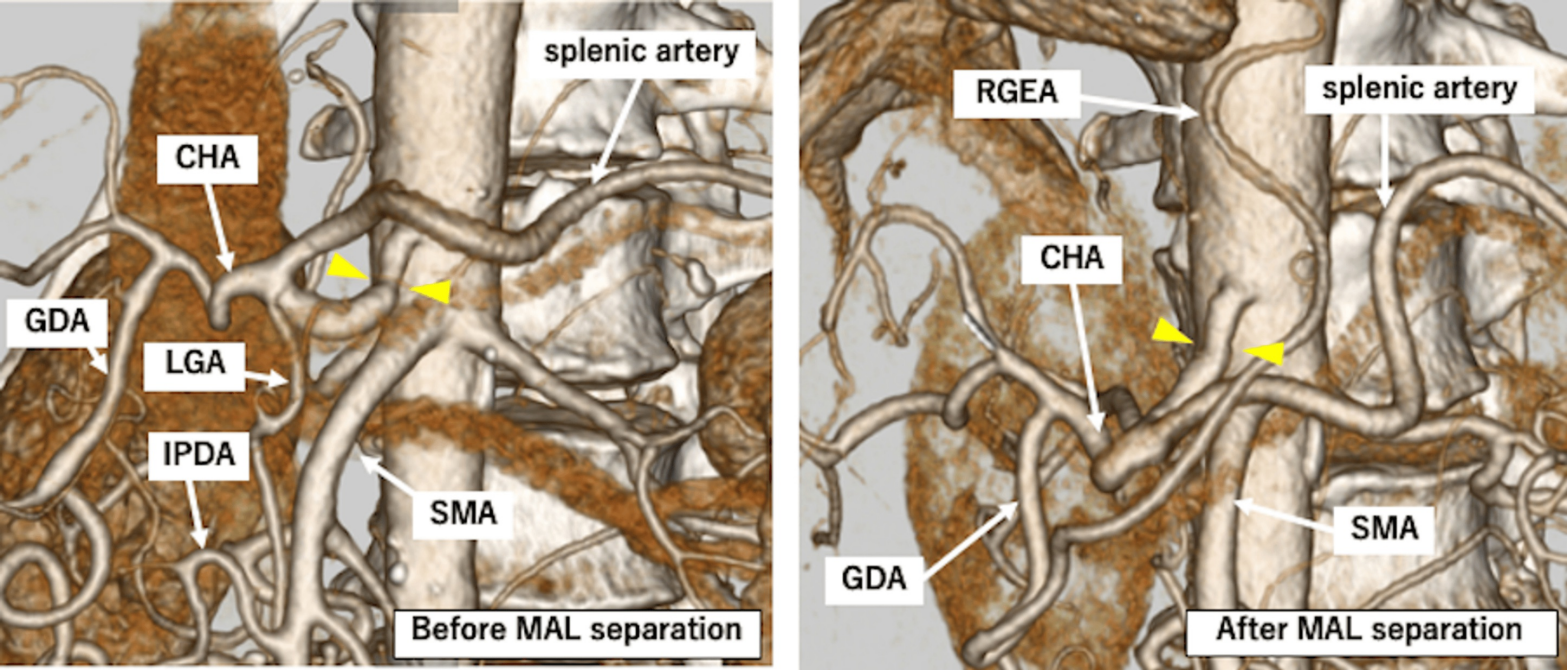
Contrast-enhanced CT of the thorax and abdomen. 3D-CT: (▲) CT images before and after MAL resection are shown. No calcification is present in the CA. (MAL: median arcuate ligament, CA: celiac artery, SMA: superior mesenteric artery, CHA: common hepatic artery, GDA: gastroduodenal artery, LGA: left gastric artery, IPDA: inferior pancreaticoduodenal artery, RGEA: right gastroepiploic artery)

Upper gastrointestinal endoscopy revealed an irregular depressed lesion extending to 1/3 of the circumference of the esophagus, 40 cm from the incisors. Abnormal vessels suggesting type B2 or higher were identified, raising suspicion of esophageal cancer with invasion beyond the submucosa. Biopsy revealed solid proliferation of atypical squamous epithelium, with prominent cellular pleomorphism and atypia, suggesting poorly differentiated squamous cell carcinoma. No detectable esophageal lesions, significant lymph node enlargement, or distant metastasis were found on chest and abdominal contrast-enhanced computed tomography (CT). Severe stenosis at the origin of the CA, probably due to the MAL, was observed on CT (Figures 1-2). No calcification of the aorta or CA was noted. The development of the pancreaticoduodenal arcade was observed, without apparent aneurysm formation.

Based on these findings, the patient was diagnosed with thoracic esophageal cancer cT1bN0M0 Stage I complicated by CACS due to MAL. A treatment plan entailing subtotal esophagectomy with gastric tube reconstruction and laparoscopic MAL release was established.

**Figure 3:**
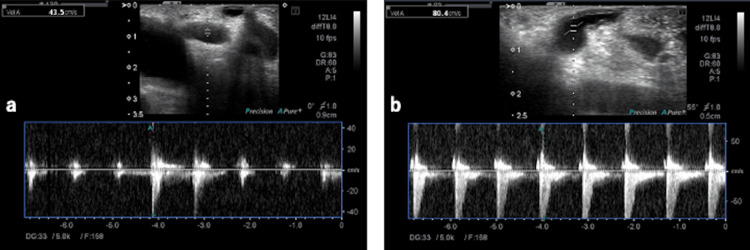
Intraoperative Doppler echocardiography. The figure shows a change in the mean flow velocity (43.5-80.4 cm/s) before and after MAL dissection, indicating that the blood flow is supplied with a more constant rhythm after dissection than that before dissection (MAL: median arcuate ligament)

**Figure 4:**
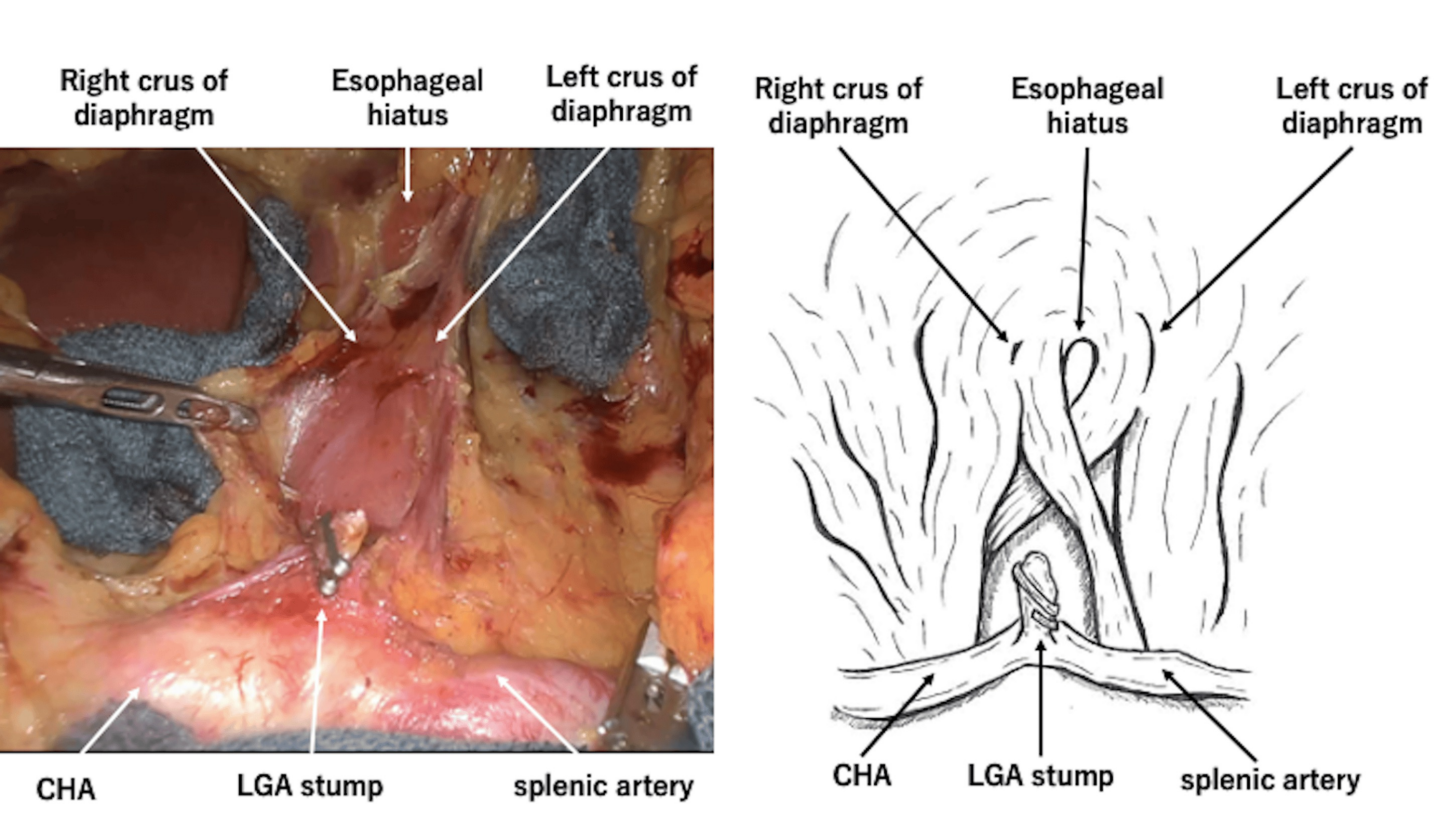
Intraoperative photographs of median arcuate ligament dissection. After abdominal lymph node dissection. The common hepatic and splenic arteries are exposed. The figure is the author's original work (CHA: common hepatic artery, LGA: left gastric artery)

**Figure 5:**
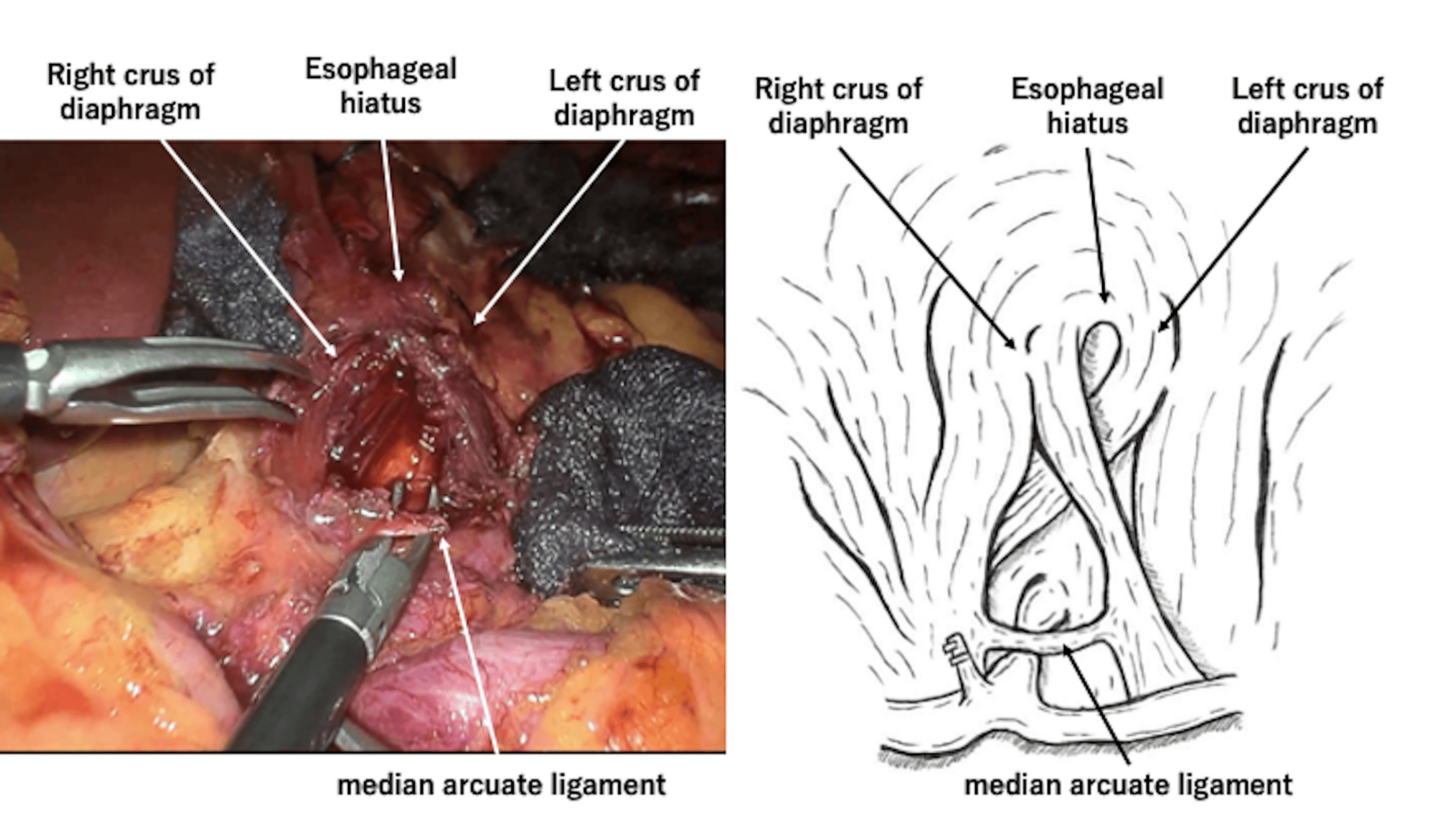
Intraoperative photographs of median arcuate ligament dissection. After MAL identification, the aortic hiatus was opened to expose the anterior aspect of the descending aorta, and the structure covering the origin of the CA was identified as the MAL. The MAL was dissected through a laparoscopic approach. The figure is the author's original work (MAL: median arcuate ligament, CA: celiac artery)

**Figure 6:**
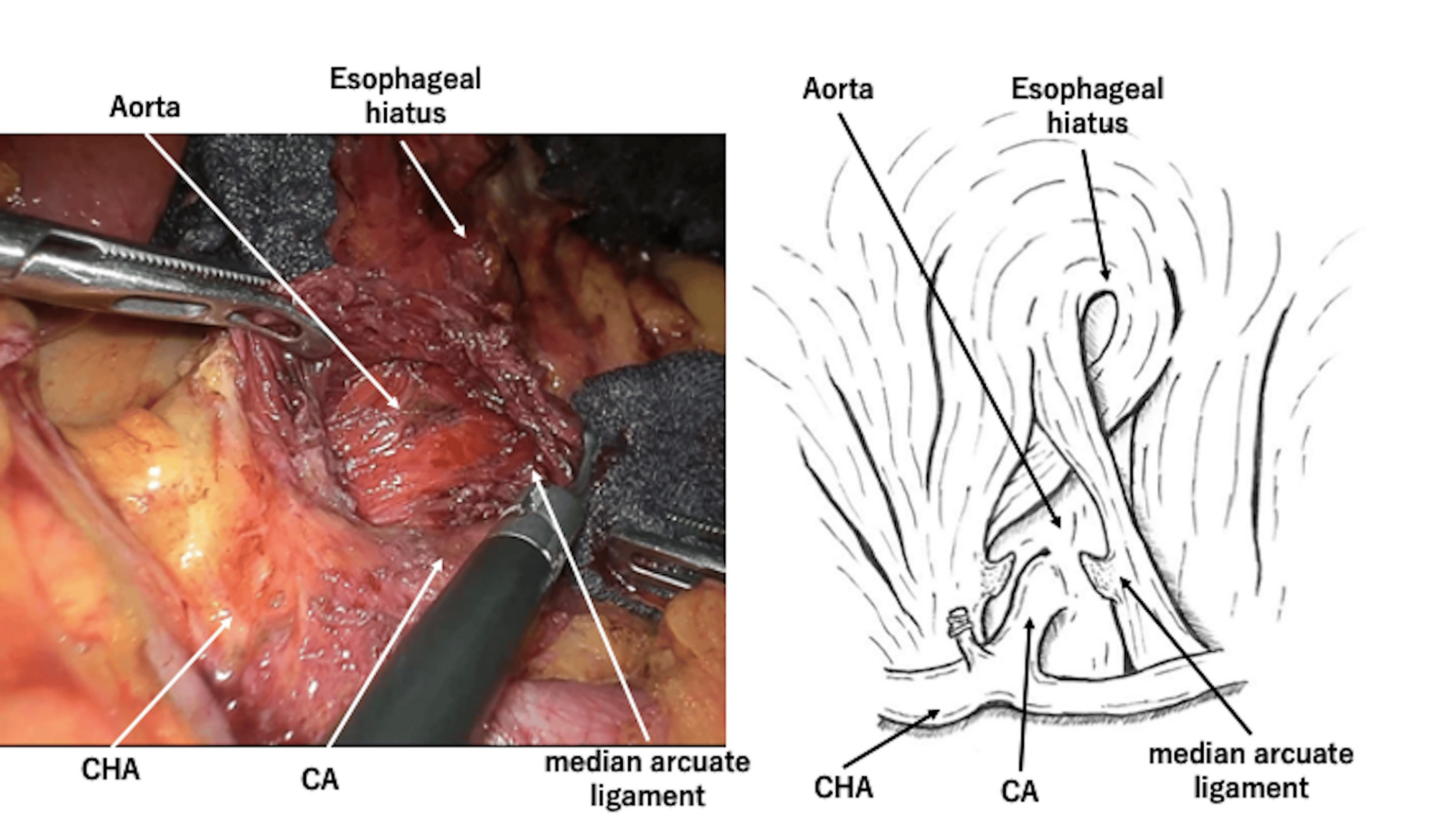
Intraoperative photographs of median arcuate ligament dissection. After dissection of the MAL, the CA was exposed to the aortic root. The figure is the author's original work (MAL: median arcuate ligament, CA: celiac artery)

The thoracic procedure was performed in the prone, and the abdominal procedure in the supine position. Lymph nodes #8a-9 were dissected, and severe stenosis at the root of the CA due to MAL was observed. Reduced blood flow velocity in the CA was confirmed using an ultrasound probe (Figure 3a). Dissection of the anterior surface of the CA and release of the MAL with an ultrasonic coagulation incision device enlarged the aortic hiatus and exposed the anterior surface of the abdominal aorta (Figures 4-6). The increase in blood flow was confirmed using an ultrasound probe (Figure 3b). A gastric tube was created with an automatic suturing device, and esophagogastric anastomosis was performed at the neck.

Histopathological examination revealed well to moderately differentiated squamous cell carcinoma (with predominance of the moderate type over the well-differentiated morphology), invading the deeper portion of the esophageal submucosa Lt, characterized by pT1b-SM2 (about 1,750 µm), Lylb, VO, INFb, pPM0, pDM0, pRM0, and IM0, cementing the diagnosis of esophageal squamous cell carcinoma type 0-lIc.

One metastatic node was detected in station 110 among the dissected lymph nodes. Anastomotic leakage was noted on postoperative day seven. Conservative treatment resulted in improvement, and the patient was discharged home on postoperative day 52.

## Discussion

Most cases of CACS are benign and asymptomatic. The etiology is variable, with atherosclerosis accounting for about 42% of cases in Japan, followed by compression by the MAL at approximately 29%. Other reported causes include aortic aneurysm syndrome, CA thrombosis/embolism, malignant lymphoma, lymph node metastasis from malignancies, and segmental arterial dissection. Narrowly defined MALS refers to a condition where the thickening of the MAL compresses and narrows the CA origin, causing various upper abdominal symptoms (postprandial angina-like abdominal pain, chronic abdominal pain, and malabsorption). Asymptomatic cases are designated as broadly defined MALS. The MAL is a tissue covering the anterior surface of the aorta and cranial to the origin of the CA and is considered a part of the diaphragmatic crura. Its composition varies among individuals, from ligamentous to unformed connective tissue. Younger women often have a closer CA-MAL relationship, and MALS is more common in slim women aged 30-50 years. Imaging and autopsy studies have shown that CA stenosis occurs in 10%-24% of patients, remaining asymptomatic in some cases. Common complaints among symptomatic patients include upper abdominal pain at rest, chronic abdominal pain, nausea/vomiting, diarrhea, unintentional weight loss, and abdominal bloating, all of which vary in severity [4]. Additionally, chronic abdominal pain, nausea, and weight loss induced by meals are observed. These symptoms are thought to result from reduced blood flow due to CA root compression, where blood flow in the CA-perfused area is supplemented via the superior mesenteric artery and gastroduodenal artery, leading to a “steal phenomenon” in the superior mesenteric artery perfusion area. MALS is a diagnosis of exclusion and should be considered in young female patients with unexplained abdominal pain. However, in this case, the patient was an elderly man who remained asymptomatic.

Although CT is useful for the imaging diagnosis of MALS, it is possible to miss conventional cross-sectional images. Thin-slice images, reconstructed coronal/sagittal images, and 3D-CT images are considered useful. One characteristic CT sign of MALS is the “hooked appearance” at the CA origin, indicating compression of the CA from the cranial direction by the MAL [5]. In this case, the development of the pancreaticoduodenal arcade suggested MALS, which was confirmed by sagittal and 3D-CT. Magnetic resonance imaging (MRI) is also considered useful for the diagnosis of MALS, and akin to CT, the CA origin exhibits the characteristic “hooked appearance.” Studies have also reported the utility of Doppler ultrasound of the CA for screening [6]. In the evaluation of CA blood flow by abdominal ultrasound, an increase in the peak systolic velocity (PSV) at the CA origin during maximum inspiration or expiration (> 200 cm/s), interruption of blood flow, or retrograde flow from the common hepatic artery is indicative of significant stenosis [7]. In a study of 46 cases undergoing ligament release surgery, the PSV at the CA origin improved from an average of 381 cm/s to 235 cm/s [8]. Typically, the product of cross-sectional area and flow velocity remains constant so that, as the vessel diameter narrows, the flow velocity increases, and as the vessel diameter widens, the flow velocity decreases. One indicator of severity is the presence of post-stenotic dilatation distal to the stenosis caused by the MAL, which can be construed as severe stenosis indicative of MALS [5]. According to intraoperative Doppler ultrasound performed before and after MAL release in this patient, the average flow velocity increased from 43.5 cm/s to 80.4 cm/s, suggesting that the pre-release measurement may have reflected post-stenotic dilatation in severe stenosis, which was alleviated by release of the MAL, leading to an increase in the flow velocity. In this case, MAL release was performed laparoscopically rather than via open surgery. Therefore, the intraoperative flow velocity was measured using a laparoscopic ultrasound probe, and the complexity of the procedure may have influenced the results. In addition to flow velocity, comparing the rhythm of pulsations before and after MAL release suggests that a more stable blood flow supply was achieved post-release.

Scholbach et al. [4] proposed the following diagnostic criteria for MALS using Doppler ultrasound: (1) the presence of postprandial symptoms, (2) vascular bruit, (3) baseline CA blood flow exceeding 200 cm/s, and (4) a decrease in CA blood flow during inspiration by more than 50 cm/s compared to baseline. They reported that 1.7% of patients with abdominal pain (n=3449, age: 0-18 years) met these criteria. The pulsatility index (PI) can be used to evaluate vascular resistance using intraoperative Doppler ultrasound [9]. It is defined as the difference between systolic and diastolic flow velocities divided by the mean flow velocity. In this case, the PI of the CA increased slightly from 0.11 to 0.18 before and after MAL release, respectively, but is thought to reflect the effect of post-stenotic dilation and cannot be conclusively interpreted as an increase in vascular resistance.

Sugae et al. [10] discussed MALS and pancreaticoduodenectomy. They evaluated CA stenosis due to MAL compression using 3D-CT images and formulated a classification based on the degree and length of stenosis: type A (less than 50%, less than 3 mm), type B (50-80%, 3-8 mm), and type C (80-100%, more than 8 mm). They concluded that MAL release is necessary during pancreaticoduodenectomy for moderate stenosis (type B) and that collateral preservation or arterial reconstruction is required for severe stenosis (type C). This case corresponds to type B or type C, supporting the presence of severe stenosis with post-stenotic dilation mentioned earlier. Colapinto et al. [11] reported a classification of CACS severity based on IVR. They assessed 152 cases of CACS by examining the degree of CA stenosis and the extent of perfusion in the CA region from the superior mesenteric artery. They classified them into grade 1 (partial perfusion of the proper hepatic artery via the pancreaticoduodenal artery), grade 2 (perfusion up to the peripheral hepatic artery), grade 3 (perfusion up to the common hepatic or left gastric artery), and grade 4 (perfusion up to the peripheral splenic artery). Additionally, they reported that almost no CA region perfusion was observed in cases with normal to 50% stenosis, while about 70% of cases with 50-75% stenosis showed grade 2 or higher results, and about 90% of cases with more than 75% severe stenosis corresponded to grades 3-4. Although IVR evaluation was not conducted in this case, the presence of post-stenotic dilation suggests severe stenosis, likely corresponding to grades 3-4.

**Figure 7:**
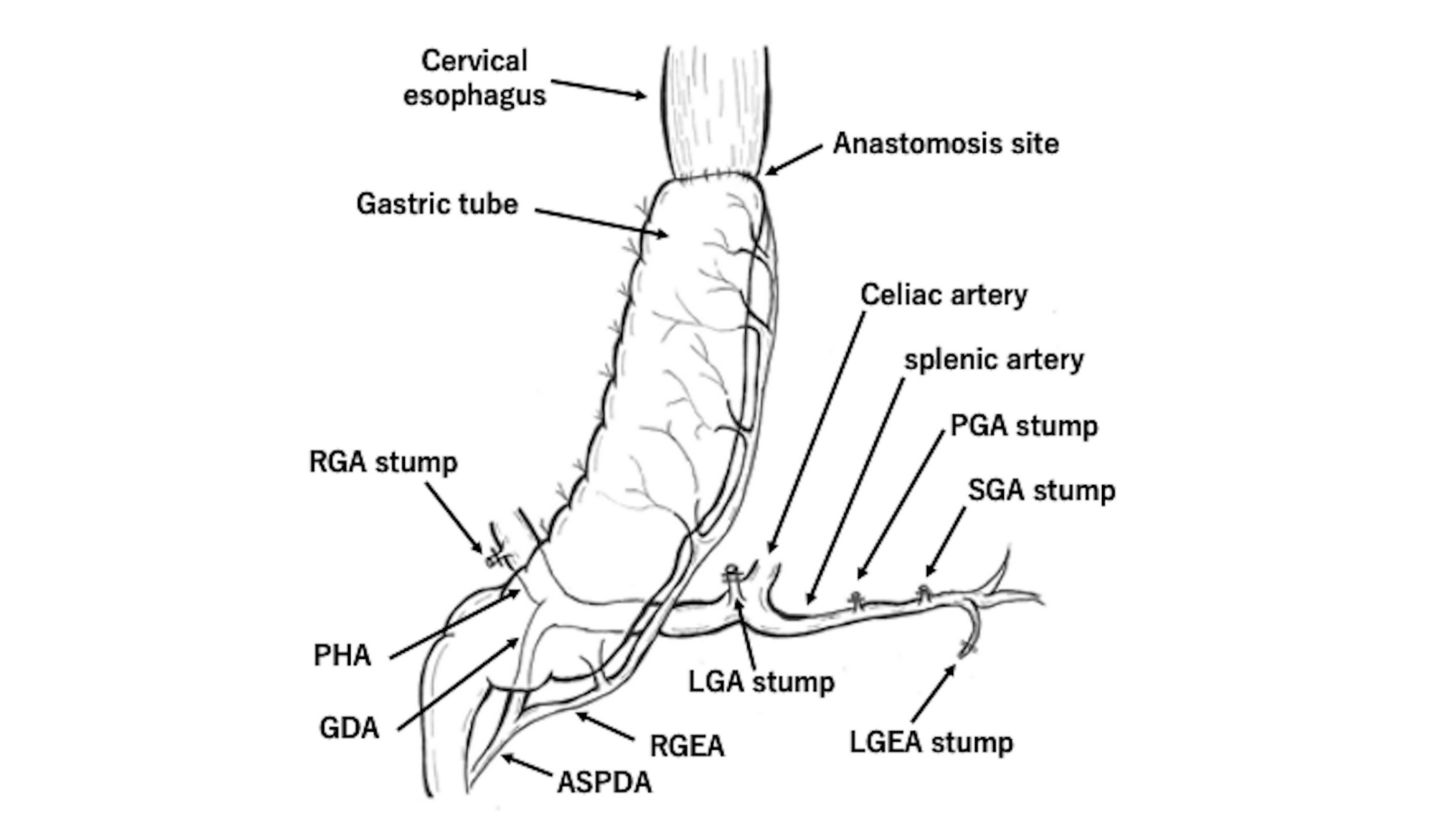
Gastric tube creation diagram. During the creation of the gastric tube, all nutrient vessels, except the right gastroepiploic artery, should be dissected. The stomach is trimmed and anastomosed with the cervical esophagus to reconstruct the gastric tube. (RGA: right gastric artery, PHA: proper hepatic artery, GDA: gastroduodenal artery, ASPDA: anterior superior pancreaticoduodenal artery, RGEA: right gastroepiploic artery, LGA: left gastric artery, LGEA: left gastroepiploic artery, SGA: short gastric artery, PGA: posterior gastric artery)

As mentioned earlier, compression of the CA by MALS leads to decreased blood flow in the right gastroepiploic artery, the only nutrient vessel in gastrointestinal tract reconstruction. As a result, ischemia at the anastomosis between the gastrointestinal tract and cervical esophagus may induce suture failure. The blood flow to the gastric tube depends on the right gastroepiploic artery, and arterial calcification in the vessels supplying the gastric tube has been identified as an independent risk factor for anastomotic leakage in esophagectomy with cervical anastomosis [12] (Figure 7).

Gastric tube ischemia is likely to occur in the presence of comorbidities related to atherosclerosis, such as diabetes, hypertension, arrhythmia, and decreased cardiac contractility [13]. This reconstruction method is associated with a very high incidence of anastomotic leakage at the gastric fundus, attributable to microcirculatory disturbances at the anastomotic site [14]. Lainas et al. [3] evaluated CA using CT and analyzed the correlation between MALS and anastomotic leakage. Ten (2.1%) of 481 patients with esophageal cancer who underwent Ivor Lewis surgery had anastomotic leakage, of which only one (10%) did not have CA stenosis. In contrast, 431 (91.5%) of 471 patients without anastomotic leakage did not have CA stenosis. Additionally, five (50%) of the 10 patients with anastomotic leakage had MALS, while only two (0.4%) of the 471 patients without anastomotic leakage had MALS. Moreover, they analyzed CA stenosis due to atherosclerotic disease, discovering that eight (80%) patients with anastomotic leakage had atherosclerotic CA stenosis, compared to 11 (2.3%) patients without anastomotic leakage. These results indicate a significant association between CA stenosis and anastomotic leakage. As mentioned previously, studies suggest that MALS contributes to the occurrence of anastomotic leakage, but there is no consensus on the time point when intervention becomes necessary. If CA blood flow is decreased, considering MAL release is appropriate. Open surgery for MAL was first reported by Dunbar [15] in 1965, and the first laparoscopic MAL release was reported in 2000 [16]. Since then, less invasive approaches have been preferred. However, due to the rarity of MALS, a standard laparoscopic MAL release procedure has not yet been established. Complete decompression of the CA is recommended, and some studies suggest that simple MAL release is insufficient, requiring extensive resection of the periceliac nerve sheath and celiac plexus [17]. In this case, we successfully improved CA blood flow by performing laparoscopic MAL release for esophageal cancer complicated by MALS. However, Clavien-Dindo Grade IIIa anastomotic leakage was observed, suggesting that the improvement in CA blood flow might have been insufficient even after MAL release. A search of PubMed using the terms “Celiac artery stenosis” and “Esophageal cancer” revealed no previous reports of laparoscopic MAL release performed during subtotal esophagectomy and gastric tube reconstruction for thoracic esophageal cancer complicated by MALS. Although we judged that CA blood flow improved in this case, it is possible that long-term compression and organic changes in the vessels may require percutaneous angioplasty or vascular reconstruction, even after MAL release [18]. Additionally, the use of indocyanine green has been proposed to easily visualize blood flow in the reconstructed gastric tube intraoperatively [18]. Many aspects such as the criteria for therapeutic intervention, choice of surgical techniques, and post-treatment evaluation methods have not been established for MALS concomitant with esophageal cancer. According to our search, this study is the first to report the combination of laparoscopic MAL release with gastric tube creation. Further accumulation of cases is awaited to establish standard treatment strategies.

## Conclusions

Reduced CA blood flow is closely related to anastomotic leakage after esophageal cancer surgery, necessitating intraoperative MAL release in MALS-complicated cases. Laparoscopic MAL release during gastric tube creation in esophageal cancer surgery is considered safe for experienced surgeons.
